# Treatment of Surgical Brain Injury by Immune Tolerance Induced by Peripheral Intravenous Injection of Biotargeting Nanoparticles Loaded With Brain Antigens

**DOI:** 10.3389/fimmu.2019.00743

**Published:** 2019-04-05

**Authors:** Zhen Tian, Lixia Xu, Qian Chen, Ruoyang Feng, Hao Lu, Huajun Tan, Jianming Kang, Yinsong Wang, Hua Yan

**Affiliations:** ^1^Graduate School of Tianjin Medical University, Tianjin, China; ^2^Tianjin Key Laboratory of Cerebral Vascular and Neurodegenerative Diseases, Tianjin, China; ^3^Department of Neurosurgery, Tianjin Huanhu Hospital, Tianjin, China; ^4^Tianjin Key Laboratory on Technologies Enabling Development of Clinical Therapeutics and Diagnostics (Theranostics), Research Center of Basic Medical Science, School of Pharmacy, Tianjin Medical University, Tianjin, China

**Keywords:** PBAE/PLGA nanoparticles, biotargeting nanoparticles, myelin basic protein, immune tolerance, surgical brain injury

## Abstract

Once excessive, neurological disorders associated with inflammatory conditions will inevitably cause secondary inflammatory damage to brain tissue. Immunosuppressive therapy can reduce the inflammatory state, but resulting infections can expose the patient to greater risk. Using specific immune tolerance organs or tissues from the body, brain antigen immune tolerance treatment can create a minimal immune response to the brain antigens that does not excessively affect the body's immunity. However, commonly used immune tolerance treatment approaches, such as those involving the nasal, gastrointestinal mucosa, thymus or liver portal vein injections, affect the clinical conversion of the therapy due to uncertain drug absorption, or inconvenient routes of administration. If hepatic portal intravenous injections of brain antigens could be replaced by normal peripheral venous infusion, the convenience of immune tolerance treatment could certainly be greatly increased. We attempted to encapsulate brain antigens with minimally immunogenic nanomaterials, to control the sizes of nanoparticles within the range of liver Kupffer cell phagocytosis and to coat the antigens with a coating material that had an affinity for liver cells. We injected these liver drug-loaded nanomaterials via peripheral intravenous injection. With the use of microparticles with liver characteristics, the brain antigens were transported into the liver out of the detection of immune armies in the blood. This approach has been demonstrated in rat models of surgical brain injury. It has been proven that the immune tolerance of brain antigens can be accomplished by peripheral intravenous infusion to achieve the effect of treating brain trauma after operations, which simplifies the clinical operation and could elicit substantial improvements in the future.

## Introduction

The central nervous system (CNS) is commonly considered to be an immunologically privileged organ that is isolated from the immune system by the blood-brain barrier (1, 2). Disruption of the blood-brain barrier caused by a neurosurgical operation can result in an inflammatory reaction in the CNS, which is known as a surgical brain injury (SBI) (3). SBI damages the blood-brain barrier, exposes many brain antigens to the immune system, creates autoimmune attacks against the brain antigens, further aggravates brain edema, neuronal damage and neuronal apoptosis, and causes irreversible neurological deficits. After the destruction of the blood-brain barrier, immune cells, cytokines, chemokines, and other inflammatory mediators are attracted to injury sites, which induces neural excitotoxicity, oxidative stress, mitochondrial dysfunction, and increased secondary inflammation that aggravates brain damage (4). Currently, treatments for secondary inflammatory responses include dehydration to reduce the intracranial pressure, anti-inflammatory steroid hormones, neurotrophic therapies, mild hypothermia therapy, and immunosuppressive agents, which can inhibit the body's immune system, which, in turn, can easily cause infection and cancer. Therefore, the exploration of efficient, safe and accurate neuroprotection strategies has become a hotspot in modern neurosurgery. Regarding the treatment of brain injury via immune tolerance, Fu et al. stated that immune interventions can reduce edema, apoptosis and brain atrophy (5). Ayer et al. reported that nasopharyngeal mucosa can be exposed to MBP to develop mucosal tolerance to these antigens for the treatment of surgical brain injury (6). In the early stage, our team also performed oral brain antigen tests to establish immune tolerance for the treatment of traumatic brain injury (7). Regarding the application of this therapy to various diseases that require a reduction of the inflammatory state of the brain tissue in the future, oral, and nasal mucosa administration may result in difficulties in determining the absorption rate, severe patients with nasal feeding and severe brain injury patients with peptic ulcers require water fasting, and traumatic brain injuries associated with facial injuries may preclude the ability to administer drugs. Xie et al. established immune tolerance via the infusion of bone marrow to reduce rejection after liver transplantation (8). Therefore, we began to apply the idea of transplantation surgery for the thymic and liver immune tolerance approach and performed thymus injections of brain antigens to establish an immune tolerance treatment for traumatic brain injury (9). Following the injection of brain antigen into the rat thymus to establish immune tolerance, rat spleen cells were co-cultured with BV-2 microglia cells (10). All of these procedures confirmed the immune tolerance effects of the injection of brain antigen into the thymus. Subsequently, we performed a comparative study of the brain injuries induced by injections of brain antigen into the thymus and into the portal vein and found that the liver pathway was superior to the thymus pathway (1, 2). However, even with the use of minimally invasive laparoscopy to administer to the hepatic portal vein, the inconvenience of the operation remained substantial. Reducing the difficulty of this operation has become a new research goal of this project.

If the hepatic portal intravenous injection of brain antigen could be replaced by normal peripheral vein infusion, the convenience of immune tolerance treatment could certainly be greatly increased. However, the problem is that the peripheral venous infusion of brain antigen inevitably leads to blood immune cell recognition and subsequent immune sensitization. How can this problem be solved? We attempted to encapsulate the brain antigen with minimally immunogenic nanomaterials and to control the size of nanoparticles within the range of liver Kupffer cell phagocytosis via coating with a material with an affinity for liver cells. We then administered this liver drug-loaded nanomaterial via peripheral intravenous injection. Due to the use of nanoparticles with liver-associated characteristics, the brain antigens were transported into the liver and were not detected by the immune armies in the blood. This approach has been demonstrated in rat models of surgical brain injury. It has been proven that the immune tolerance of brain antigen can be achieved by peripheral intravenous infusion to achieve the effect of treating brain trauma after an operation, which simplifies the clinical operation and could elicit substantial improvements in the future.

## Results

### The Drug Loading Properties of Brain Antigen-PVA/PBAE/PLGA Nanoparticles

**Figure 1:**
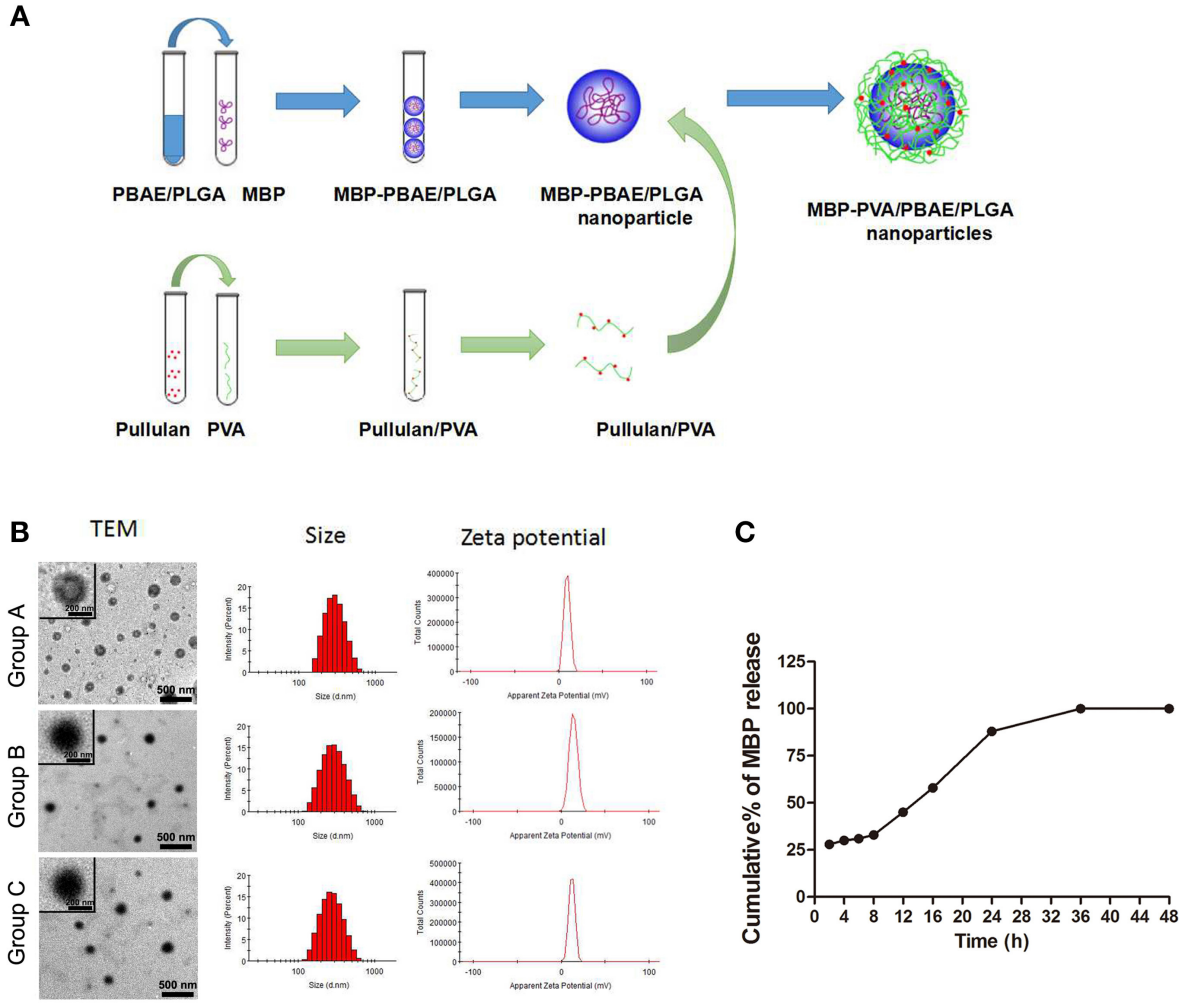
**(A)** Synthesis flow diagram of PVA/PBAE/PLGA nanoparticles loaded with MBP. **(B)** Transmission electron microscopy determinations of the particle sizes and charge characteristics of the PVA/PBAE/PLGA nanoparticles loaded with different brain antigens. Group A: PVA/PBAE/PLGA nanoparticles loaded with normal saline. Group B: PVA/PBAE/PLGA nanoparticles loaded with MBP. Group C: PVA/PBAE/PLGA nanoparticles loaded with brain proteins. **(C)** The release profiles of MBP from the PVA/PBAE/PLGA nanoparticles.

Scheme of MBP-PVA/PBAE/PLGA nanoparticles prepared was shown in Figure 1A. The concentrations of MBP and brain protein were measured by ultraviolet spectrophotometry to calculate the drug loading and encapsulation ratio. The particle sizes of the nanoparticles loaded with MBP and brain protein were 269.9 and 272.2 nm, respectively. The PDI of each nanoparticle was < 0.2, which indicated that the particle size distribution was uniform. The zeta potentials were 13.2 and 12.1 mV, respectively. The encapsulation ratios of the proteins in the two groups were 64.5 and 62.4%, respectively (Table 1). TEM revealed that the PVA/PBAE/PLGA/MBP and PVA/PBAE/PLGA/brain protein nanoparticles exhibited regular sphere and typical “core-shell” structures (Figure 1B). And we depicted the release profiles of MBP from the PVA/PBAE/PLGA nanoparticles. The quantity of MBP released was followed ultraviolet spectrophotometry at 280 nm. The release of MBP was observed up to 36 h (Figure 1C).

**Table 1:** Characteristics of the PVA/PBAE/PLGA nanoparticles loaded with different antigens.

**Drug-PVA/PBAE/PLGA (w-o-w)**	**Size^**a**^ (d/nm)**	**PDI^**a**^ (mV)**	**Zeta potential**	**EE^**b**^ (%)**
NS	291.3	0.261	9.5	/
MBP	269.9	0.142	13.2	64.5%
Protein	272.2	0.176	12.1	62.4%

a *The size (mean diameter) and polydispersity index (PDI) were determined via the dynamic laser light scattering method at least three times*.

b *Drug encapsulation efficiency (EE) was defined as the weight percentage ratio between the loaded drug and the added drug during preparation*.

### Distribution of the PVA/PBAE/PLGA Nanoparticles in Nude Mice

**Figure 2:**
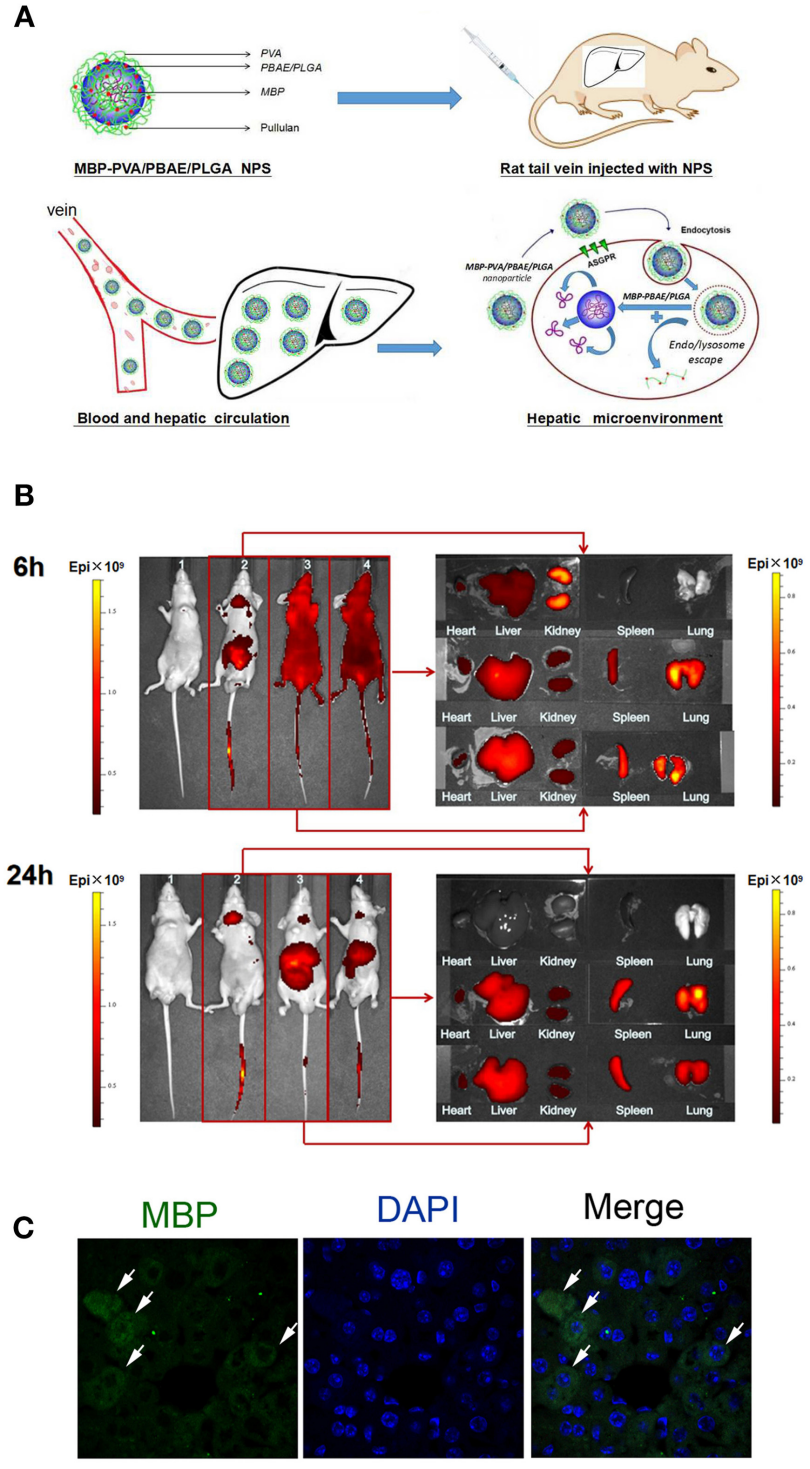
**(A)** Schematic diagram of the structure of MBP-PVA/PBAE/PLGA nanoparticles and the release mechanism of nanoparticles in the liver. **(B)** Exploration of the distributions of free Cy5.5, PVA/PBAE/PLGA/Cy5.5, and PBAE/PLGA/Cy5.5 nanoparticles in nude mice organs at 6 and 24 h. Small animals were imaged *in vivo* (1: saline-injected group; 2: free Cy5.5-injected group; 3: PVA/PBAE/PLGA/Cy5.5 nanoparticle-injected group 4: PBAE/PLGA/Cy5.5 nanoparticle-injected group). **(C)** Immunofluorescent staining of MBP in PVA/PBAE/PLGA/MBP nanoparticles-treated liver.

The schematic PVA/PBAE/PLGA/MBP nanoparticles structure diagram was shown in Figure 2A. Detection of the distribution and accumulation of PVA/PBAE/PLGA nanoparticles in nude mice allowed for the evaluation of the biological targeting of the liver. PVA/PBAE/PLGA nanoparticles were labeled with the near-infrared fluorescent dye Cy5.5 and then injected into mice via the tail vein. The mice injected with free Cy5.5 were observed after 6 h, and the liver's red fluorescence signal was weak, whereas the kidney's red fluorescence signal was stronger. The PVA/PBAE/PLGA/Cy5.5 and PBAE/PLGA/Cy5.5 nanoparticles were distributed throughout the blood vessels after 6 h of administration and began to accumulate in the liver. The mice were dissected to observe the main organs, and the free Cy5.5 was found to be mainly distributed in the kidney, with small amounts appearing the liver and heart. The PVA/PBAE/PLGA/Cy5.5 and PBAE/PLGA/Cy5.5 nanoparticles were mainly distributed in the liver and spleen, and only a few were distributed in the kidney, heart, and lung tissues. The mice injected with free Cy5.5 exhibited complete elimination of the dye after 24 h, while the mice injected with PVA/PBAE/PLGA/Cy5.5 nanoparticles exhibited accumulation mainly localized to the liver after 24 h. The mice were dissected, and the main organs were observed. The free Cy5.5 was found to be completely removed from all organs, whereas the PVA/PBAE/PLGA/Cy5.5 and PBAE/PLGA/Cy5.5 nanoparticles were mainly distributed in the liver tissues. The following pictures clearly demonstrated that the distribution of free Cy5.5 was very short in the liver, and the dye was rapidly removed from the mice. The PVA/PBAE/PLGA/Cy5.5 and PBAE/PLGA/Cy5.5 nanoparticles exhibited obvious liver targeting. The accumulation time and concentration in the liver were significantly prolonged, and the clearance time in the mice was significantly delayed (Figure 2B). Thus, the use of the PVA/PBAE/PLGA nanoparticle surface modification in this experiment changed the tissue distribution of the drugs in the mice and significantly improved the accumulation of drug release in the liver. To confirm whether the PVA/PBAE/PLGA/MBP nanoparticles can be engulfed in liver, we stained the liver sections with antibody against MBP by immunofluorescent staining and found that nanoparticles were engulfed in phagocytes in the liver (Figure 2C).

### Co-culture of T Lymphocytes and BV-2 Microglia Cells

**Figure 3:**
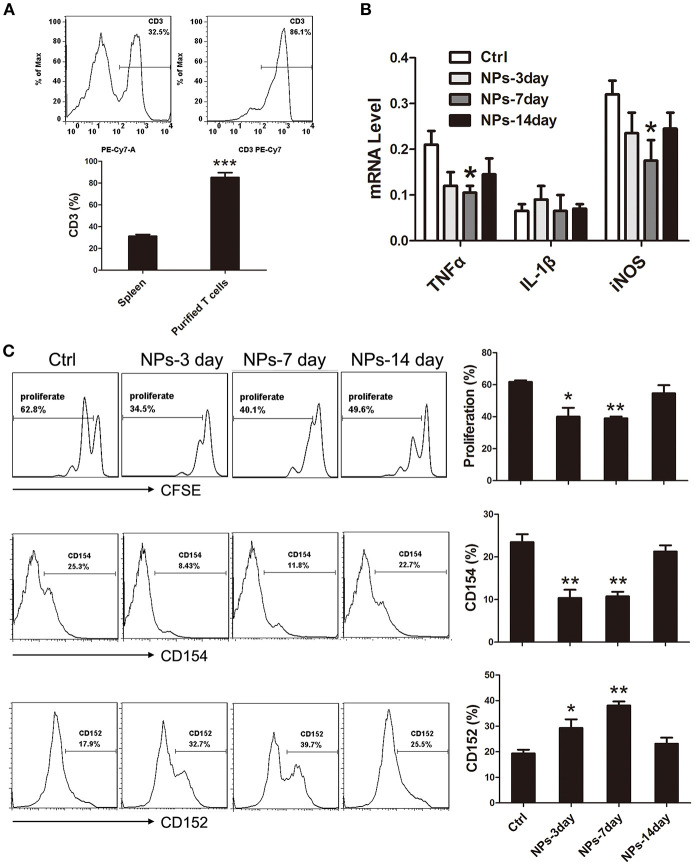
**(A)** Extraction of T lymphocytes from nanoparticles-treated mice. **(B)** Expressions of pro-inflammatory factors of BV-2 microglia cells (IL-1β, TNF-α, and iNOS). **(C)** FACS analysis of CFSE and CD152, CD154 in cultured T cells. *compared with Ctrl, *P* < 0.05, **compared with Ctrl, *P* < 0.01.

To further confirm whether peripheral intravenous injection of PVA/PBAE/PLGA/MBP nanoparticles in mice could induce immune tolerance to brain antigen, we derived T lymphocytes from the spleens of nanoparticles treated mice at postoperative days 3, 7, and 14, respectively, and then co-cultured with activated BV-2 microglia cells at a proportion of 1:2 in medium containing MBP for 3 days. Figure 3A showed the percentage of T lymphocytes extracted from spleen could reach 85.0 ± 5.0%. After 3 days of co-culture, we first detected the expressions of pro-inflammatory factors of BV-2 microglia cells (IL-1β, TNF-α, and iNOS), and found that the expressions of TNF-α and iNOS in 7-day nanoparticles-treated mice were significantly lower than those in no-treated mice (Figure 3B). Next, we performed CFSE staining for proliferation of T cells, and found that the proliferation of MBP-specific T cells was significantly decreased after the nanoparticles intravenous injections as compared with the no-treated mice (Figure 3C). Last, we analyzed the expression of surface antigens of CD152 (CTLA4, immune checkpoint inhibitor, downregulating immune responses) and CD154 (CD40L, primarily expressed on activated T cells) in T lymphocytes by flow cytometry, and found that the levels of CD152 in nanoparticles-treated mice, especially 7 days, were more abundant than that in no-treated mice, by the contrast, the levels of CD154 in nanoparticles-treated mice were decreased (Figure 3C).

### Neurological Function Scoring

**Figure 4:**
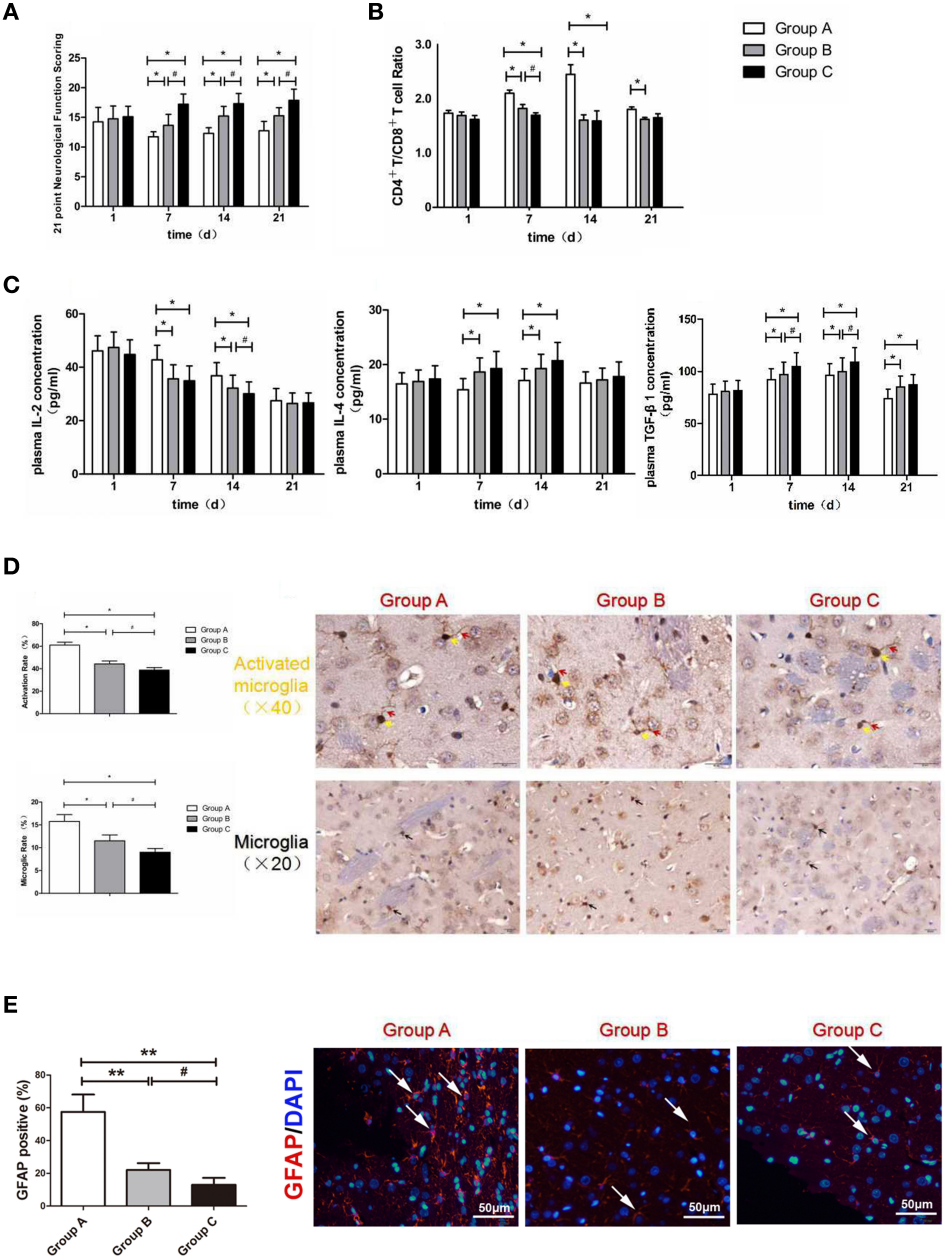
**(A)** Neurological function scoring histogram. **(B)** Histogram of peripheral blood CD4^+^T/CD8^+^T cell ratios. **(C)** Histogram of the proinflammatory cytokine IL-2, IL-4, and TGF-β1.*compared with Group A, *P* < 0.05; ^#^compared with Group B, *P* < 0.05. **(D)** Iba-1 expression scoring of microglial cells measured at 21 d after SBI. *compared with Group A, *P* < 0.05; ^#^compared with Group B, *P* < 0.05. The brown-positive cells are activated microglial cells, the yellow arrows indicate the somata of the activated microglia, and the red arrows indicate the processes of the activated microglia (x40). The black arrows indicate positive results (the brown cells) (x20). The scale bars are 20 μm. **(E)** GFAP expression scoring of astrocytes measured at 21 d after SBI. *compared with Group A, *P* < 0.05, **compared with Group A, *P* < 0.01; ^#^icompared with Group B, *P* < 0.05. The scale bars are 50 μm.

There were different degrees of neurological dysfunction in all groups after SBI. The neurological scores were notably increased in Group B and Group C compared with Group A at 7, 14, and 21 d after SBI (*P* < 0.05). Additionally, the neurological scores in Group C were higher than those in Group B at 7, 14, and 21 d after SBI (*P* < 0.05; Figure 4A). The above results demonstrated that the nanoparticles loaded with brain protein performed better than the nanoparticles loaded with MBP in terms of promoting neurological scores.

### CD4^+^/CD8^+^ T Cell Ratios in the Peripheral Blood

T cells serve as a vital part of the immune system, specifically CD4+T and CD8+T cells. Yilmaz et al. (11) found that cerebral ischemia/reperfusion injury significantly increased the level of CD4+T cells in cerebral tissue, whereas it decreased the level of CD8+T cells. Another study also demonstrated that the CD4^+^T/CD8^+^T cell ratio increased in cerebral ischemia/reperfusion injury sites (12). Compared with Group A, the CD4^+^T/CD8^+^T cell ratios were markedly decreased postoperatively in Groups B and C at 7 and 14 d (*P* < 0.05). Moreover, there was significant difference between Group B and Group C on the 7th day (*P* < 0.05; Figure 4B). The above results demonstrated that the intravenous injection of nanoparticles loaded with MBP and the intravenous injection of nanoparticles loaded with brain protein were able to decrease the CD4^+^T/CD8^+^T cell ratios; moreover, the nanoparticles loaded with brain protein were better than the nanoparticles loaded with MBP in terms of decreasing the CD4^+^T/CD8^+^T cell ratio.

### Concentrations of the Pro-inflammatory Cytokine IL-2 in the Peripheral Blood

Compared with Group A, the IL-2 concentrations were markedly decreased postoperatively in Group B and Group C at 7 and 14 d (*P* < 0.05). Moreover, there was significant difference between Group B and Group C on the 14th day (*P* < 0.05; Figure 4C). The above results demonstrated that the intravenous injection of nanoparticles loaded with MBP and the intravenous injection of nanoparticles loaded with brain protein were able to decrease the pro-inflammatory cytokine IL-2 concentration. Moreover, nanoparticles loaded with brain protein were superior to the nanoparticles loaded with MBP in terms of decreasing the IL-2 concentration.

### Concentrations of the anti-inflammatory Cytokine IL-4 in the Peripheral Blood

Compared with Group A, the IL-4 concentrations were markedly increased postoperatively in Group B and in Group C at 7 and 14 d (*P* < 0.05). The above results demonstrated that the intravenous injection of nanoparticles loaded with MBP and the intravenous injection of nanoparticles loaded with brain protein were able to increase the anti-inflammatory cytokine IL-4 concentrations. However, the nanoparticles loaded with brain protein and the nanoparticles loaded with MBP did not show differences in reducing IL-4 expression.

### Concentrations of the Inflammation-Suppressing Cytokine TGF-β1 in the Peripheral Blood

Compared with Group A, the TGF-β1 concentrations were markedly increased postoperatively in Groups B and C at 7 and 14 d (*P* < 0.05). Moreover, there was significant difference between Group B and Group C on the 7th and 14th days (*P* < 0.05; Figure 4C). The above results demonstrated that the intravenous injection of nanoparticles loaded with MBP and brain protein could increase TGF-β1 concentrations. Moreover, nanoparticles loaded with brain protein were better than nanoparticles loaded with MBP in terms of increasing the TGF-β1 concentrations.

### Microglia iba-1 Immunohistochemistry

Microglia are important glial cells of the central nervous system. Microglia are also the first and foremost immunological defense in the central nervous system. After traumatic brain injury, microglia can rapidly divide, effectively remove the dying cell debris in the damaged area, reshape the injured brain tissue, and prevent neuronal damage. The morphologies of microglia are generally divided into branched and amoeba-like. Normally, microglia are in a resting state. Microglia have the characteristics of a small cell body and long branching processes and continuously monitor the microenvironment around the central nervous system through the function of neurons. When central nervous system damage causes a secondary inflammatory response, stimulated microglia are rapidly activated, the morphological manifestations of the cell bodies become larger, and the protrusions are retracted in an amoeba-like fashion, which plays an important role in phagocytosis (13). This high degree of morphological plasticity exhibited by microglia, which involves the rapid transformation from quiescent branched cells to amoebic cells, is also known as the “functional plasticity” process (14). Therefore, we can determine whether microglia are activated by changes in their morphologies (Figure 4D).

Ionized calcium binding adaptor molecule-1 (iba-1) is a 17-kDa EF chiral protein that is a specific calcium binding protein of microglia and participates in membrane fold formation and phagocytosis in activated microglia. Miyamoto et al. (15) found that after traumatic brain injury, the microglia surface antigen marker iba-1 protein expression increases; therefore, the detection of the expression of this protein can reflect the degree of microglial activation. Our experimental results revealed that the microglial iba-1 activation rate and ratios in Group B and Group C were significantly lower than those in Group A (*P* < 0.05). Compared with Group B, the microglial iba-1 activation rate and ratio in Group C were significantly lower (*P* < 0.05). Thus, the injection of nanoparticles loaded with MBP or brain protein into the tail veins of rats was able to significantly reduce the number of microglia and decrease the proportion of activated microglia. Compared with the MBP-loaded nanoparticles, the brain protein-loaded nanoparticles significantly reduced iba-1 expression in activated microglia and thereby reduced the nerve damage due to secondary inflammation.

### Astrocytes GFAP Immunofluorescence

Astrocytes play a crucial role in inflammation, tissue repair and glial scar formation after brain injury, and have the potential to improve the sequelae of brain injury. We determined the expression of GFAP in the surrounding peri-resection brain samples by immunofluorescence staining (Figure 4E). We found that GFAP expression in Group B and Group C were significantly lower than those in Group A (*P* < 0.05), which was similar with the Iba-1 results, indicated that the injection of nanoparticles loaded with MBP or brain protein was able to significantly reduce the activation of astrocytes.

### Nerve Cell FasL Expression Immunohistochemistry

**Figure 5:**
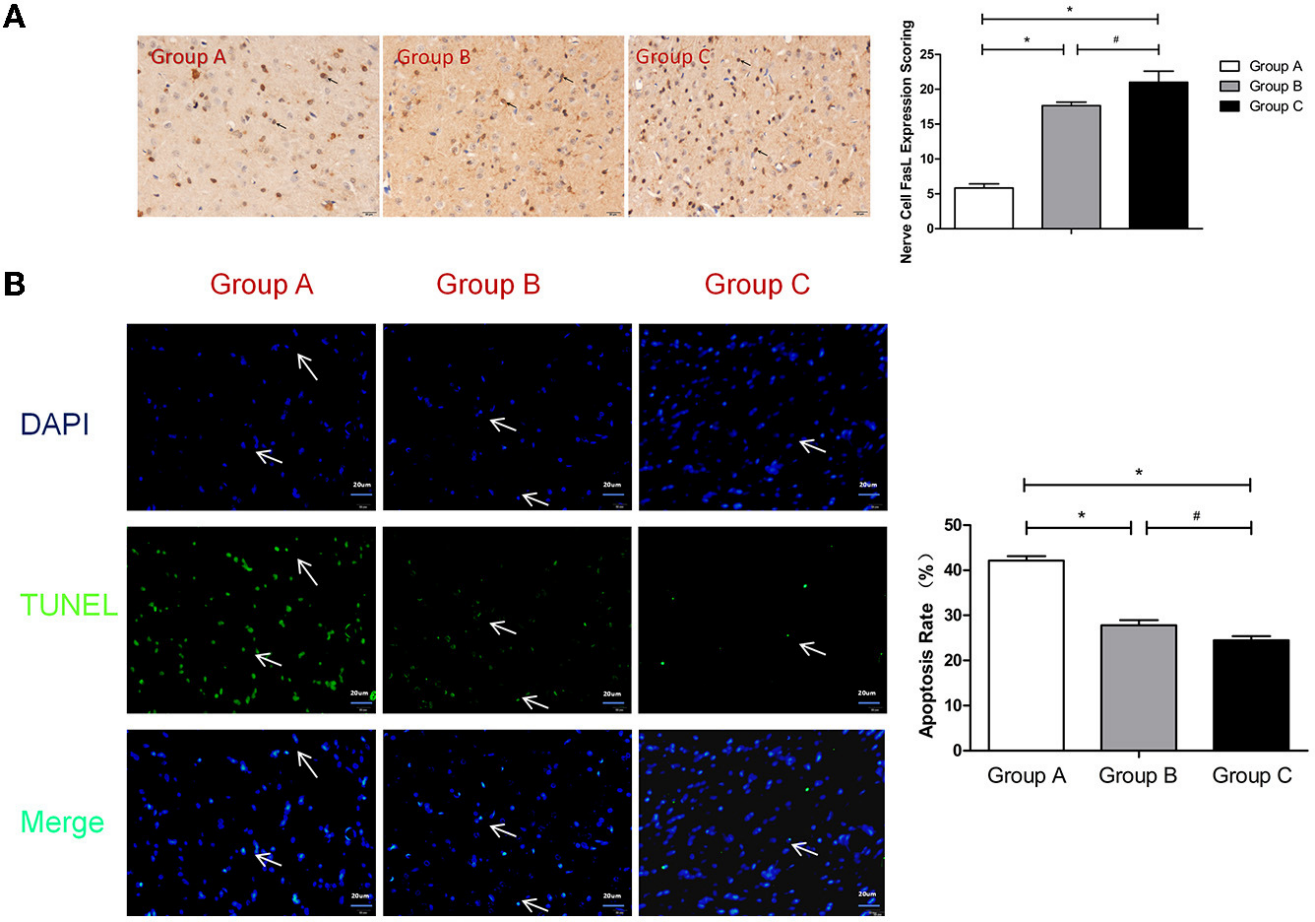
**(A)** FasL expression scoring of nerve cells measured at 21 d after SBI. *compared with Group A, *P* < 0.05; ^#^compared with Group B, *P* < 0.05. The black arrows indicate positive results (the brown cells). **(B)** TUNEL assay of the nerve cells. *compared with Group A, *P* < 0.05; #compared with Group B, *P* < 0.05. DAPI (blue) was used to indicate the nuclei (arrows), and TUNEL (green) was used to indicate the apoptotic signals (arrows). The merge indicates the apoptotic cells (arrows). The scale bars are 20 μm.

Fas is composed of a cytoplasmic domain, a transmembrane domain and an extracellular domain. The extracellular amino acid composition is relatively conserved and mainly plays the role of a receptor that initiates apoptotic signaling after ligand binding. Therefore, the Fas antigen is a death molecule. Fas Ligand (FasL) is a type-II transmembrane protein that belongs to the tumor necrosis factor (TNF) family that can specifically bind to Fas on target cell membranes and induce apoptosis. Quiescent T cells express low levels of Fas, but these levels can be significantly up-regulated after activation. Additionally, FasL exhibits slight expression in the immune-privileged organ of the brain. FasL induces the apoptosis of immune cells through the Fas/FasL pathway, which protects the brain tissue from immune damage (16). Akiyama et al. found that the bone marrow mesenchymal stem cells expressed in the whole body can exhibit FasL-induced Fas-expressing T lymphocyte apoptosis (17). Our experimental results demonstrated that the neuronal FasL expression levels in Group B and Group C were significantly higher than that in Group A (*P* < 0.05). Compared with Group B, Group C exhibited much greater promotion of nerve cell expression of FasL (*P* < 0.05; Figure 5A). Thus, the injection of nanoparticles loaded with MBP or brain protein into the tail veins of rats induced a significant increase in the expression of FasL, and nanoparticles loaded with brain protein promoted the expression of FasL more significantly in nerve cells and thereby reduced nerve damage due to secondary inflammation.

### TUNEL Assay of the Nerve Cells

TUNEL detection is a commonly used method for detecting the apoptosis of brain cells (18). Our experiment revealed that the apoptosis rates in Group B and Group C were significantly lower than that in Group A (*P* < 0.05). Compared with Group B, the apoptosis rate of Group C was significantly lower (*P* < 0.05; Figure 5B). Thus, the injection of nanoparticles loaded with MBP or brain protein into the tail veins of rats induced a significant decrease in the apoptosis rate, and the nanoparticles loaded with brain protein were superior to the nanoparticles loaded with MBP in terms of decreasing nerve cells apoptosis.

## Discussion

Brain trauma, neurosurgery, spontaneous cerebrospinal meningitis, etc. will expose brain antigens to the immune system due to the destruction of the blood-brain barrier, which leads to an attack by the autoimmune system that further aggravates brain tissue damage. Immunosuppressive therapy has been demonstrated to exhibit therapeutic effects, but infection and long-term tumor progression, as well as a narrow drug safety spectrum, limit its clinical promotion. Reducing the immune tolerance against brain antigen treatment has become a hot spot. In 1946, Chase et al. (19) found that antigens reduced the body's reactivity to antigens through oral administration or portal vein route infusion, and could induce the body's specific tolerance to antigens, so the liver was considered as an organ of immune preference. In 1969, Calne first found that, in the absence of immunosuppression, the main histocompatibility complex did not match the transplanted liver antigen that could be tolerated by the host (20). Subsequently, immune tolerance has received increasing attention. Huang et al. found that injecting bone marrow mesenchymal stem cells through the thymus and vein can prolong the survival of transplanted rat hearts (21). Immune tolerance has been clinically applied in organ transplantation. Yang et al. found that immune tolerance can be established by transplanting hematopoietic stem cells to reduce the immune response after pancreas transplantation (22). Vanikar et al. co-infused mesenchymal stem cells and hematopoietic stem cells for renal transplantation immune tolerance (23). How immune tolerance therapy with brain antigen can be clinically applied is a problem that urgently needs to be solved. As the most advanced nerve center, the brain is not only structurally specific but also tends to directly affect the use of the administration route once affected. We have already enumerated the restrictive conditions, such as water fasts, facial injuries, etc.; therefore, the thymus and liver have become the new choices. Because we have confirmed that the liver pathway is superior to the thymus pathway, further developing the convenience of the liver pathway has become a new goal. If liver portal vein injection can be replaced by peripheral vein injection, the result would not only be non-invasive but also reduce the difficulty of the administration to nearly nothing, which is the project's main technical innovation.

Because the liver is the body's largest antigen-removing organ, any foreign molecules, especially particulate antigen material, will be cleared by Kupffer cells if they have the opportunity to pass through the liver. This clearance is also the process by which Kupffer cells act as immune antigen-presenting cells to establish immune tolerance. Of course, smaller particles or antigens can also be cleared by endosomal sinusoidal endothelial cells to complete immune tolerance presentation. The liver is an indispensable “chemical plant” of the body. It has the function of biotransformation, which can transform foreign substances with immune response into non-immune response substances required by the body. If brain antigen not through the liver transformation directly into the blood circulation will produce immune enhancement and even immune attack. We hypothesized that brain antigen could be encapsulated with liver-targeted nanobiological material. After infusion into a peripheral vein, the brain antigen could be enriched and retained in the liver by blood circulation and nanomaterial liver targeting (which takes full advantage of the active and passive targeting of particles). The establishment of immune tolerance by immune antigen-presenting Kupffer cells and sinusoidal endothelial cells not only achieves convenient antigen delivery but also achieves the highly efficient utilization of a small amount of precious antigen. This approach also avoids the immunological sensitization caused by conventional peripheral antigen injection and successfully avoids the monitoring of open blood immune cells.

Passively targeted administration, or naturally targeted administration, means that the drug-carrying particles are taken up by the mononuclear macrophages (especially liver Kupffer cells) and transported to the liver, spleen, and other organs through normal physiological processes (24). The particle concentration of the drug in the liver can greatly reduce the systemic drug distribution and dose, as well as the number of administrations, improve the drug treatment index, and reduce adverse reactions. After intravenous injection, the distribution of passively targeted particles in the body depends on the size of the particles. Particles with sizes larger than 7 μm are usually trapped by mechanical filtration through the smallest capillary bed in the lungs and are taken up by mononuclear cells into the lung tissue or into the lungs. Particles <7 μm are generally ingested by macrophages in the liver and spleen. Nanoparticles of 200–400 nm are concentrated in the liver and rapidly engulfed by Kupffer cells, whereas nanoparticles smaller than 10 nm are slowly accumulated in the bone marrow (25).

In this experiment, the biological targeting nanomaterials were the key that enabled not only the efficient loading of brain proteins but also a high degree of liver targeting and good blood stability. We designed nanoparticles as a vector for brain antigens, and their consortium resulted in strong liver targeting and accumulation properties. PBAE as a novel kind of cationic polymers firstly developed by Langer's group (26). It has a strong “proton-sponge” effect, resulting in the breakdown of endosomes/lysosomes and the intracellular release of the carried protein, however, due to the poor stability of PBAE in blood circulation, it is easily swallowed by mononuclear macrophages (27). This experiment solves these problems through the following methods. Firstly, poly (lactic co glycolic acid) (PLGA) was added to the PBAE nanoparticles to enhance its stability and regulate the release efficiency of the protein; secondly, with branched chain pullulan on the surface of PBAE/PLGA nanoparticles (pullulan) polyvinyl alcohol (PVA) coated with a layer of film, in order to protect the positive charge (28). Many studies have confirmed that surface modification is the best strategy for changing the stability of PBAE's drug carriers (29). PVA/PBAE/PLGA nanoparticles can be efficiently taken up by hepatocytes through endocytosis mediated by the asialoglycoprotein receptor (ASGPR) (30) and ASGPR is widely expressed on the surface of hepatic parenchymal cells. In addition, due to the negative charge of the cell membrane, the positive charge nanoparticles can efficiently enter the liver cells through the endocytic pathway mediated by charge interactions, and the intracellular endosomes/lysosomes induce PBAE “proton-sponge” effect, resulting in the breakdown of endosomes/lysosomes and the intracellular release of carried brain proteins. Therefore, the PVA/PBAE/PLGA nanoparticles prepared in this study can effectively uptake hepatocytes through endocytosis mediated by asialoglycoprotein receptor (ASGPR) and the interaction between charges, and then through the PBAE/PLGA “proton-sponge” effect to achieve efficient release of brain protein within hepatic parenchymal cells.

Any material assembled *in vitro* must be preliminarily verified by *in vivo* animal experiments. Using a rat model of brain injury, we evaluated the behavioral scores associated with the use of new drugs for animal brain injury, the indexes of blood immune tolerance, and the degree of brain cell injury and even conducted a systematic assessment of the activation of key immune cells, i.e., microglia, in the brain tissue. Finally, we confirmed that peripheral venous transfusion, the most commonly clinically used route, was able to establish the immune tolerance of brain antigens and reduce the secondary immune damage to brain tissue. Of course, the key to this technology is the endowment of the brain antigens with excellent “masquerading” and physical characteristics suitable for phagocytosis in the liver, as well as navigation that provides “guiding” function. Although the preparation of *in vitro* drug particles is slightly complicated, the technology for the preparation of nanomaterials can be identified in the modern pharmaceutical industry, and the convenience brought by clinical promotion represents a breakthrough.

The content of MBP in the central nervous system is second only to myelin protein lipoprotein (MPLP), which accounts for about 30% of the total myelin protein. Central MBP is synthesized and secreted by oligodendrocytes, which is the major brain antigen component, while mixed brain tissue proteins contain multiple antigens (31). In the case of rat tail vein injections with equal protein loading, the loading with mixed brain antigens was superior to that with nanoparticles loaded with the single brain antigen MBP, and the tail vein injection of mixed brain protein-loaded nanoparticles induced immune tolerance more effectively than the injection of single MBP-loaded nanoparticles, thus alleviating damage due to secondary inflammation. The reason for this difference is that brain protein is a mixture of multiple brain antigens that can elicit the synergistic effect of bystander inhibitory effects (32). Bystander inhibitory effect was activated by and tolerogen resulted in the suppression of immune responses to other antigens. Another study found that experimental arthritis can be prevented by mucosal administration of microbial peptide via bystander suppression. The bystander inhibitory effect is the process of tolerating the immune response of the original immune system to other antigens (33). Therefore, the rat tail vein injection of nanoparticles loaded with brain protein for the treatment of secondary inflammation was superior to that of nanoparticles loaded with MBP. In conclusion, the effect of loading mixed brain proteins was superior to that of loading MBP alone.

In recent years, the approaches utilized to induce immune tolerance have mainly focused on oral induction (34, 35), nasal mucosal instillation (3), thymus injection (9), and liver injection (1). Although these methods have achieved some efficacy in clinical studies and animal experiments, some problems still remain: (1) Oral induction and nasal mucosal instillation depend on mucosal absorption, so the absorption rate and the amount of absorbed antigen cannot be controlled. Additionally, most patients with severe brain injury are unconscious and thus have poor compliance, and common complications, such as stress ulcers, also affect the intestinal mucosa in terms of antigen processing function. (2) After traumatic brain injury, water fasts, injuries to the nasal complex, rhinitis, etc. limit nasal mucosal tolerance treatment. (3) Thymus injection-based immune tolerance requires thymus puncture under ultrasound guidance, so anesthesia problems, positioning failures, operational errors, and other risks are present. (4) Hepatic portal vein injection requires minimally invasive laparoscopic surgery in general surgery departments, which not only increases the trauma to the patient but also increases the probabilities of infection and surgical complications. With the introduction and promotion of the concept of precision medicine and multidisciplinary convergence in recent years, our group hopes to propose a new technology that is not limited by the route of administration and causes minimal additional damage to the patient. The peripheral intravenous injection of nanoparticles loaded with brain antigen initially seems to meet the above requirements. This work has developed a new method for the treatment of secondary brain inflammation caused by brain injury and other diseases that require reduction of the inflammation of brain tissue in clinical neurosurgery and lays the experimental foundation for clinical transformation.

## Methods

### Repeated Freeze-Thaw Method to Extract the Brain Protein

Intraperitoneal injection of 0.3 ml/100 g 10% of chloral hydrochloride anesthesia, after total anesthesia, the skin was exposed. Fix the rats on stereotactic apparatus and quickly broke neck, on aseptic conditions and sagittal view cut the scalp, separate skull, expose brain tissue and remove the brain tissue, then carefully remove small blood vessels and meningeal tissue on the surface, cut up brain tissue on aseptic condition, quickly place into −80°C for 30 min and 37°C water bath for 5 min to rewarm quickly, join rod-shaped grinding mill for full grinding under the condition of 4°C, repeat this step for three to five times, using the inverted phase contrast microscope to confirm there was no brain cellular morphology, test protein concentration by using Ultraviolet spectrophotometry, configure to solution of 1.0 mg/ml concentration with ultrapure water, place in −20°C (36).

### Preparation and Characterization of Brain Antigen-PVA/PBAE/PLGA Nanoparticles

The nanoparticles were prepared according to the programme provided by professor Wang Yinsong of the project team (37, 38). First, the PBAE/PLGA nanoparticles loaded with brain protein were prepared by means of emulsion solvent volatilization as follows: 4 mg of beta poly β-amino ester (PBAE) and 20 mg of poly lactic-co-glycolic acid (PLGA) (mass ratio) 1/5, a total of 24 mg are dissolved in 1 ml methylene chloride (CH_2_Cl_2_), after immersion in 100 μl MBP (1.0 mg/ml) (Cat: 09011680, Enzolifescience) or 100 μl brain protein (1.0 mg/ml), 4°C under the condition of ice bath kept stirring, by ultrasonic cell crushing apparatus 200 W ultrasonic emulsification process to its 3 min, form a water/oil (W/O) nano emulsion, namely for emulsifying PBAE/PLGA nanoparticles. Then, the surface of the PBAE/PLGA nanonuclei will be modified by PVA, which contains a pullulan. Method is as follows: 100 mg of polyvinyl alcohol (PVA) and 100 mg of Pullulan polysaccharides (Pullulan) dissolved in 10 ml pH7.4 slightly ultrapure water won 1% of the polyvinyl alcohol (PVA), to the preparation of PBAE/PLGA colostrum (W/O) add to 4 ml of 1% polyvinyl alcohol (PVA) solution in the 4°C under the condition of ice bath kept stirring, by ultrasonic cell crushing apparatus 200 W ultrasonic emulsification process to its 3 min, form a water/oil/water (W/O/W) emulsion, join the 5 ml 2% isopropyl alcohol solution, and under the room temperature ventilated lasts 800 RPM stir 2 h to remove methylene chloride (CH_2_Cl_2_), then placed in a centrifuge 10,000 RPM (4°C) after 10 min, take the supernatant by ultraviolet spectrophotometric instrument measuring the concentration of MBP to calculate the envelopment rate. Nanoparticle dispersed again after repeated centrifugation, washing in ultrapure water after the heavy suspension emulsion stored at 4°C, won the emulsifying loading brain protein of PVA/PBAE/PLGA nanoparticles. Brain antigen- PVA/PBAE/PLGA nanoparticles with the concentration of 0.5 mg/mL scattered in ultrapure water, using ultraviolet spectrophotometry at 240 nm and 300 nm wavelength detection PVA/PBAE/PLGA/MBP and PVA/PBAE/PLGA/brain protein nanoparticles in detecting the concentration of MBP and brain protein computing drug-polymer interactions and coating rate. The particle size and particle size distribution were detected by dynamic laser scattering method, zeta potential was detected by zeta potentiometer, and the surface charge property was investigated. The morphology of nanoparticles was observed by TEM.

### *In vitro* Release of PVA/PBAE/PLGA Nanoparticles

The *in vitro* release profile of the MBP-loaded PVA/PBAE/PLGA nanoparticles was observed at 37°C in phosphate-buffered saline medium. The quantity of MBP released was followed spectrophotometrically at 280 nm.

### Distribution of PVA/PBAE/PLGA Nanoparticles in Nude Mice

The liver-biotargeting capability of PVA/PBAE/PLGA nanoparticles was analyzed by *in vivo* bioluminescence imaging. PVA/PBAE/PLGA nanoparticles were carried on the Cy5.5 near-infrared fluorescence markers, and the tissue distribution of nanoparticles in mice was investigated by using the IVIS *in vivo* imaging system (39). Typically, sixteen nude mice (Experimental Animal Center of Academy of Military Medical Sciences, China) were randomly separated into four groups (*n* = four per group) and were injected with normal saline (control), free Cy5.5, PVA/PBAE/PLGA/Cy5.5 and PBAE/PLGA/Cy5.5 nanoparticles via tail vein, and then imaged using IVIS *in vivo* imaging system (PerkinElmer, USA) at 6 and 24 h post the injection point in time, and main organs (heart, liver, kidney, spleen, and lung) observed and photographed using IVIS *in vivo* imaging system, to evaluate the distribution of nanoparticles in nude mice.

### MBP Immunofluorescence

The capability of PVA/PBAE/PLGA/MBP nanoparticles engulfed in liver was analyzed by immunofluorescence staining. PVA/PBAE/PLGA nanoparticles were carried on the MBP protein, rats were injected with PVA/PBAE/PLGA/MBP nanoparticles via tail vein. Twelve hours after injected with nanoparticles, rats were anesthetized and sacrificed by perfusing through the heart with 4% paraformaldehyde in PBS (pH 7.4). Liver was removed, postfixed overnight and sectioned by the Department of Pathology. The sections were pretreated with xylene and slides into the liquid sodium citrate repair water bath heating repair, then add the MBP antibodies(Cat: ab62631, Abcam) in the slides, at 4°C overnight. Then they were incubated with Andy FluorTM 488-Streptavidin staining solution and counterstained with DAPI(Cat: ZLI-9557, Zhongshan). Finally, they were covered with antifade mounting medium. Images were captured by a fluorescence microscopy (Olympus). MBP kit, DAPI and antifade mounting medium were purchased from GeneCopoeia Inc(Sigma USA).

### SBI Model Design and Rat Tail Intravenous Injection of Brain Antigen PVA/PBAE/PLGA Nanoparticles

All methods were performed in accordance with the relevant guidelines and regulations and approved by the Tianjin Key Laboratory of Cerebral Vascular and Neurodegenerative Diseases, China. Laws and rules were strictly obeyed to protect the animals from abuse. Twenty-four male Sprague-Dawley rats (grade SPF; 200–220 g) (Experimental Animal Center of Academy of Military Medical Sciences, China) were randomly divided into three groups with eight rats per group. A standardized SBI model was used according to previous reports (40). Briefly, before surgery, general anesthesia was induced via intraperitoneal injection of 0.3 ml/100 g chloral hydrate. The head and manubrium sterni areas of the rats were shaved. Next, each rat was fixed in a stereotaxic frame and the sterile skin on the skull was incised along the biparietal suture through a single sagittal incision. A small square of skull (4 mm diameter) was thinned and removed with a bone drill on the right skull bone 2 mm along the sagittal suture and 1 mm along coronal suture. A durotomy was performed, and 2 × 3 mm brain tissue was excised by sharp dissection. Hemostasis was confirmed, and then the skin was closed. Immediately after SBI, the nanoparticles loaded with normal saline, MBP or brain protein were, respectively, injected into the tail vein of Group A, B, and C.

### Extraction of T lymphocytes

Every mouse in each group was sacrificed to obtain the spleen according to the method in Gottrand et al.'s study (41) at postoperative days 3, 7, and 14, respectively. Before the sacrificion, nylon fiber column (Polysciences, PA, USA) was incubated for 1 h using 1,640 culture medium containing 10% fetal bovine serum (FBS) (Gibco, MA, USA). The mice were sacrificed by carbon dioxide inhalation and then immersed in 75% alcohol for 10 min to be sterilized. Their spleens were obtained and grinded on 200-mesh stencil aseptically. Grinding fluid was centrifuged in 900 rpm for 5 min, the supernatant was abandoned, and 2 ml 1X red blood cell lysis buffer was added. The solution was then centrifuged again, the supernatant was abandoned, the deposit was resuspended by PBS, and then, cells were collected into the culture flask after transiting nylon fiber columns.

### Co-culture of BV-2 Cells and T lymphocytes

Interferon-γ (IFN-γ) (PeproTech, NJ, USA) (100 U/ml) and granulocyte macrophage-colony stimulating factor (GM-CSF) (PeproTech, NJ, USA) (10 ng/ml) were added to BV-2 cell culture medium to activate BV-2 cells for 7 days before co-culture. Isolated T-cells were added to flasks which stayed in horizontal position for 3 h. Then, the flasks were stood upright in order to wipe off monocytes. Interleukin (IL)-2 and ConA were needed in co-culture of BV-2 cells and T-cells. Afterward, T-cells were added to the glial culture medium containing MBP in the proportion of 1:2 (0.5 × 10^5^ T-cells: 1 × 10^5^ BV-2 cells).

### CFSE for Proliferation

The freshly purified T cells (10^7^cells/ml) were labeled with CFSE (5 μM) (BD bioscience)for 10 min at 37°C. Labeling was quenched with RPMI 1,640 supplemented with 10% FCS and the cells were washed twice before co-culturing with BV-2. FACS analysis was performed after 72 h of incubation.

### Detection of Surface Antigens by Flow Cytometry

The antibodies were offered by BD Biosciences (CA, USA). After identified T lymphocytes by anti-CD3(Cat:552774, BD bioscience), anti-CD152(Cat:564331, BD bioscience), anti-CD154(Cat:561719, BD bioscience), and their isotype controls were applied to T-cells.

### Quantitative Real-Time Polymerase Chain Reaction (PCR) for Pro-inflammatory Factors

The expressions of pro-inflammation genes of BV-2 microglia cells, including tumor necrosis factor-α (TNF)-α, IL-1β, and inducible nitric oxide synthase (iNOS), were detected by quantitative real-time PCR. M-MLV kit was bought from Life Technology Inc. (USA), regarding microglia RNA as template reverse transcription. into complementary DNA. The primers (Sigma, USA) used were as follows:

* TNF-α: Forward 5′-CATCTTCTCAAAATTCGAGTGACAA-3′, reverse 5′-TGGGAGTAGACAAGGTACAACCC-3′;
* IL-1β: Forward 5′-CAACCAACAAGTGATATTCTCCATG-3′, reverse 5′-GATCCACACTCTCCAGCTGCA-3′;
* iNOS: Forward 5′-CAGCTGGGCTGTACAAACCTT-3′, reverse 5′-CATTGGAAGTGAAGCGTTTCG-3′;
* GAPDH:Forward 5′-TTCACCACCATGGAGAAGGC-3′, reverse 5′- GGCATGGACTGTGGTCATGA -3′.

### Neurological Function Scoring

An independent researcher who was blinded to experimental designs practiced the modified Garcia scoring system. This scoring system was used to evaluate neurological function in the rats at 1, 7, 14, and 21 d after surgery, as previously described. Briefly, the maximum score of this scoring system is 21 points based on sensorimotor performances ranging from 0 to 3 for the following seven areas: spontaneous activity, side stroking response, vibrissae response, limb symmetry when the tail was suspended, lateral turning when the tail was suspended, symmetry of walking on the forelimbs when the tail was partially suspended, and climbing ability/response. Neurological scores were assigned as follows: 0 = complete deficit, 1 = definite deficit with some function, 2 = mild deficit or decreased response, and 3 = no evidence of deficit/symmetrical responses. The maximum score on this scale is 21 points, representing normal neurological function.

### Serum Cytokine Concentrations

After induction of general anesthesia, rats were fixed with supine position and blood sampling by cardiac puncture was used to sample 1 ml blood that was injected into promoting coagulation tube. Followed by 30 min standing at room temperature, the blood sample was centrifuged with 3,500 rpm/min, 15 min. The serum was shifted to EP tube and stored at −80°C. According to the ELISA kit protocol (Boster Biological Technology, Wuhan, China) plus the TMB color drops, then proinflammatory factor IL-2, anti-inflammatory factor IL-4 and inflammation suppression factor TGF-β1 concentrations were measured at 1, 7, 14, and 21 d postoperatively by using the enzyme reader at 450 nm.

### Flow Cytometry

The protocol of above mentioned blood sampling was as follows. All antibodies were purchased from BioLegend. Antibodies were directly marked with one of the following fluorescent tags: fluorescein isothiocyanate (FITC), phycoerythrin (PE), or allophycocyanin (APC). Anti-CD3(Cat: 552774, BD bioscience), anti-CD(Cat: 550954, BD bioscience), anti-CD8a(Cat: 557654, BD bioscience) antibodies and isotypes were used to react with rat antigens. Cell surface phenotype and sorting were recognized using a flow cytometer (BD company, USA), and the data were analyzed using FlowJo 7.6.1 software.

### Nerve Cell FasL Immunohistochemistry

Rats were anesthetized and sacrificed by perfusing through the heart with 4% paraformaldehyde in PBS (pH 7.4). Brains were removed, postfixed overnight and sectioned by the Department of Pathology, Tianjin Huanhu Hospital. The sections were pretreated with xylene and then incubated with primary antibody against rat Fas ligand (Abcam Anti-Fas Ligand antibody), at 4°C overnight. Next, sections were incubated with complement, HRP conjugate, and DAB, orderly (Abcam expose Mouse and Rabbit Specific HRP/DAB Detection IHC kit). Next, sections were counterstained with hematoxylin. Images were captured by a microscopy (Olympus).

The total FasL immunostaining score was computed according to Wang et al. (39). Briefly, the percentage of positive was defined as 0 (<5%, negative), 1 (5–25%, sporadic), 2 (25–50%, focal), or 3 (>50%, diffuse). Staining intensity was defined as 0 (no staining), 1 (weak staining), 2 (moderate staining), or 3 (strong staining). The total score of immunostaining was computed as the percentage positive score × staining intensity score, and ranged from 0 to 9. We examined five successive fields as a section and summed the scores.

### Microglia Cell Iba−1 Immunohistochemistry

Sections were prepared as above. The sections were pretreated with xylene and slides into the liquid sodium citrate repair water bath heating repair, then add the iba−1 antibodies in the slides, at 4°C overnight. Next, sections were incubated with complement, HRP conjugate, and DAB, orderly (Abcam expose Mouse and Rabbit Specific HRP/DAB Detection IHC kit). Next, sections were counterstained with hematoxylin. Images were captured by a microscopy (Olympus). The percentage of activation was calculated by the formula: activation rate (%) = positive activated microglia/all positive microglia plasma × 100%, ratio (%) = all positive cells microglia pulp/plasma × 100%.

### GFAP Immunofluorescence

Sections were prepared as above. The sections were pretreated with xylene and slides into the liquid sodium citrate repair water bath heating repair, then add the GFAP antibodies(Cat: ab7260, Abcam) in the slides, at 4°C overnight. Then they were incubated with the secondary antibody staining solution and counterstained with DAPI(Cat: ZLI-9557, Zhongshan). Finally, they were covered with antifade mounting medium. Images were captured by a fluorescence microscopy (Olympus).

### TUNEL Immunofluorescence

Sections were prepared as above. The sections were deparaffinized and rehydrated with xylene and then permeabilized with proteinase K solution. Next, the sections were reacted with TdT reaction buffer, TdT reaction cocktail, orderly. Then they were incubated with Andy FluorTM 488-Streptavidin staining solution and counterstained with DAPI. Finally, they were covered with antifade mounting medium. Images were captured by a fluorescence microscopy (Olympus). TUNEL kit, DAPI, and antifade mounting medium were purchased from GeneCopoeia Inc, Sigma Life Science, and ZSGB-BIO Company, respectively.

The percentage of apoptosis was calculated by the formula: apoptosis rate (%) = (TUNEL-positive nuclei)/(total cell number) × 100%.

### Statistical Analysis

All date in this study were presented as means ± SD. Statistical analyses were performed with SPSS software (version IBM SPSS Statistics 22.0) and cartograms were drew with GraphPad Prism software (version5.01). One-way ANOVA and LSD test were used to determine the significance of differences among subgroups. The data of the non-normal distribution used rank sum test and *P* < 0.05 was considered statistically significant.

## Author Contributions

HY designed and conceived the study, study supervision, and critical revision of the manuscript for intellectual content. YW performed the nanoparticles. JK directed the SBI model. ZT, LX, RF, HL, and HT performed the experiment. ZT and QC analyzed the data. ZT, RF, and QC wrote the manuscript. ZT, LX, and QC contributed equally to this study.

### Conflict of Interest Statement

The authors declare that the research was conducted in the absence of any commercial or financial relationships that could be construed as a potential conflict of interest.
