# Corrigendum: Treatment of surgical brain injury by immune tolerance induced by peripheral intravenous injection of biotargeting nanoparticles loaded with brain antigens

**DOI:** 10.3389/fimmu.2023.1335541

**Published:** 2023-12-08

**Authors:** Zhen Tian, Lixia Xu, Qian Chen, Ruoyang Feng, Hao Lu, Huajun Tan, Jianming Kang, Yinsong Wang, Hua Yan

**Affiliations:** ^1^ Graduate School of Tianjin Medical University, Tianjin, China; ^2^ Tianjin Key Laboratory of Cerebral Vascular and Neurodegenerative Diseases, Tianjin, China; ^3^ Department of Neurosurgery, Tianjin Huanhu Hospital, Tianjin, China; ^4^ Tianjin Key Laboratory on Technologies Enabling Development of Clinical Therapeutics and Diagnostics (Theranostics), Research Center of Basic Medical Science, School of Pharmacy, Tianjin Medical University, Tianjin, China

**Keywords:** PBAE/PLGA nanoparticles, biotargeting nanoparticles, myelin basic protein, immune tolerance, surgical brain injury

In the published article, there was an error in **Figures 4** and **5** as published. In **Figure 4D**, two images were placed in opposite positions. In **Figure 5B**, we provided a more appropriate picture. The corrected **Figures 4** and **5** and its caption appear below.

**Figure 4:**
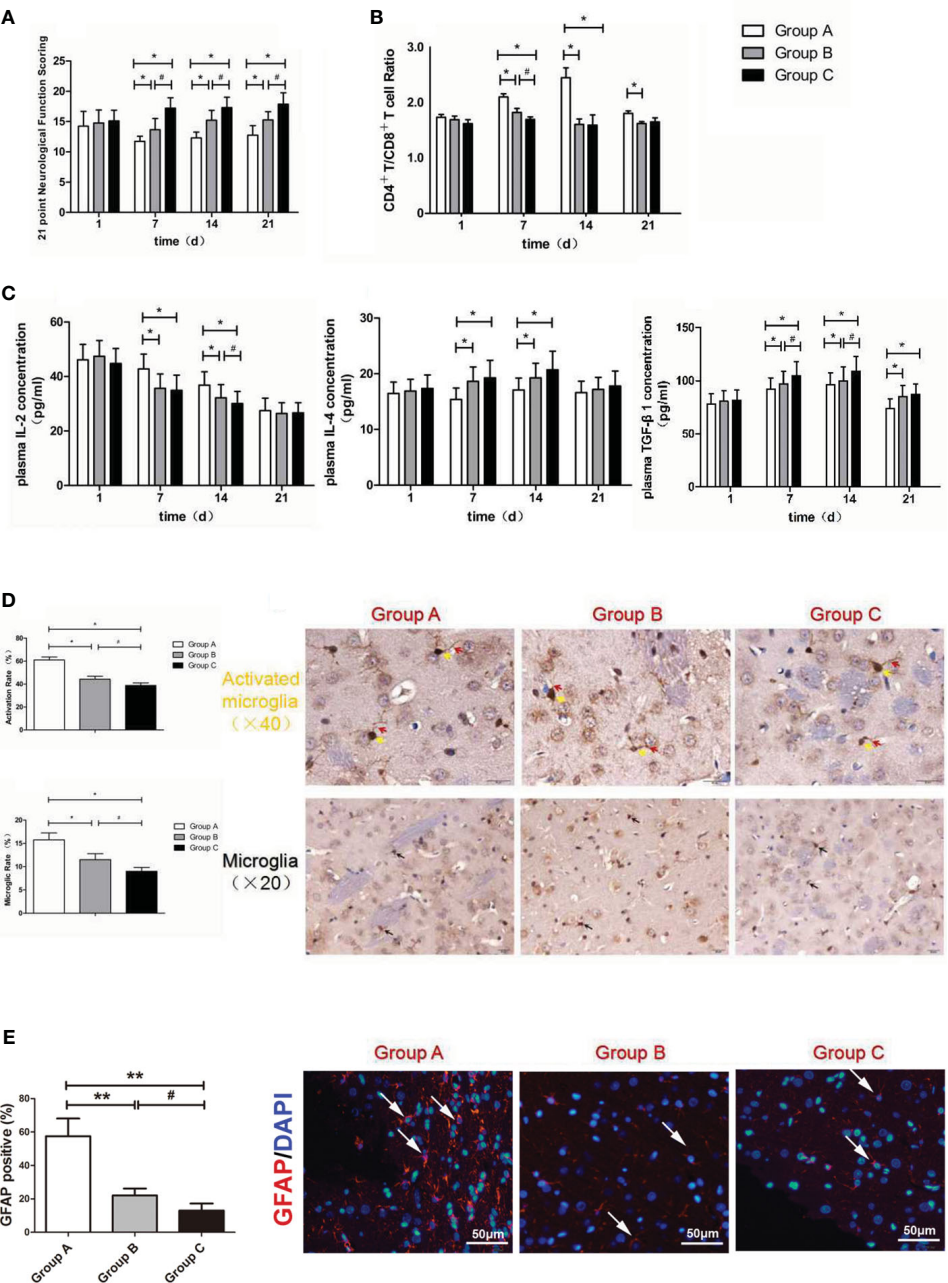
**(A)** Neurological function scoring histogram. **(B)** Histogram of peripheral blood CD4^+^T/CD8^+^T cell ratios. **(C)** Histogram of the proinflammatory cytokine IL-2, IL-4 and TGF-β1.^*^compared with Group A, P < 0.05; ^#^compared with Group B, P < 0.05. **(D)** Iba-1 expression scoring of microglial cells measured at 21 d after SBI. ^*^compared with Group A, P < 0.05; ^#^compared with Group B, P < 0.05. The brown-positive cells are activated microglial cells, the yellow arrows indicate the somata of the activated microglia, and the red arrows indicate the processes of the activated microglia (x40). The black arrows indicate positive results (the brown cells) (x20). The scale bars are 20μm. **(E)** GFAP expression scoring of astrocytes measured at 21 d after SBI. ^*^compared with Group A, P < 0.05, **compared with Group A, P < 0.01; ^#^compared with Group B, P < 0.05. The scale bars are 50μm.

**Figure 5:**
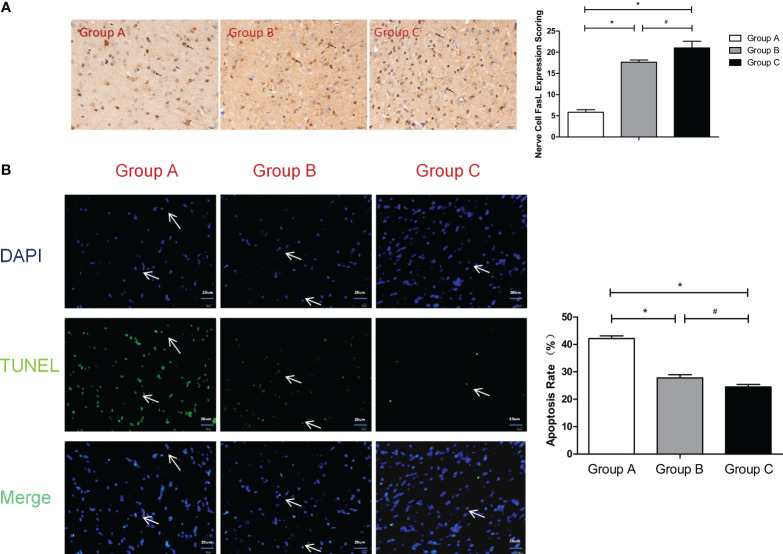
**(A)** FasL expression scoring of nerve cells measured at 21 d after SBI. *compared with Group A, P < 0.05; #compared with Group B, P < 0.05. The black arrows indicate positive results (the brown cells). **(B)** TUNEL assay of the nerve cells. *compared with Group A, P < 0.05; # compared with Group B, P < 0.05. DAPI (blue) was used to indicate the nuclei (arrows), and TUNEL (green) was used to indicate the apoptotic signals (arrows). The merge indicates the apoptotic cells (arrows). The scale bars are 20 μm.

The authors apologize for these errors and state that this does not change the scientific conclusions of the article in any way. The original article has been updated.

